# Serynges replacement for insulin application for pens device in a population of elderly patients with type 2 diabetes: multifactorial intervention to improve glycemic control

**DOI:** 10.1186/1758-5996-7-S1-A50

**Published:** 2015-11-11

**Authors:** Rafael Vaz Machry, Henrique Umpierre Pedroso, Rafaela Ramos Nunes, Luthiele da Silva Vasconcellos, Cibelle de Abreu Evaldt, Thaymê Luísa de Souza Pires, Raquel Ferreira, Eduardo Bardou Yunes Filho, Paloma Dias da Cruz, Ticiana da Costa Rodrigues

**Affiliations:** 1 Hospital De Clínicas de Porto Alegre, Porto Alegre, Brazil

## Background

It is known that better glycemic control reduces the chronic complications of diabetes (DM). The target of the glycemic control is difficult to achieve, and only 25-50% of the patients achieve the goals. Some studies show that different devices for insulin can improve the adherence.

## Objectives

Evaluate the glycemic response after changing the insulin syringes (SY) for pens device (PD) in patients chronically decompensated already in insulin use.

## Materials and methods

This is a prospective, intervention, non-randomized, phase IV study. We included patients over 60 yrs. old, both sexes, with HbA1c >8.5% using oral hypoglycemic agents and insulin and then we replaced SY by PD. We used human insulin NPH and regular as pens, all patients have received a blood glucose monitor, lancet tapes, capillary blood glucose tests (3 tests/day). HbA1c was measured at baseline, 3 and 6 months. Patients were seen monthly.

## Results

Analysis was “intention-to-treat” of the 45 patients included. HbA1c, at baseline was 10.34±0.22, similar to the values 12 and 6 months prior to inclusion. HbA1c was 8.54±0.23 and 8.09±0.21, after 3 and 6 months, respectively, with no difference among them. After 3 months of the end of study, there was a deterioration of HbA1c (9.67±0.38). Patients remained using PD. During the study, there was an increase in total daily insulin dose prescribed (0.84±0.07 to 1.06±0.10UI/kg, p<0.001) and increase in regular/NPH insulin ratio (0.12±0.02 to 1.22±0.04, p=0.001), with no increasing of BMI (31.7±0.72 vs. 32.13±0.79kg/m2, p=0.82). Moreover, we found no difference in the occurrence of hypoglycemia (p=1.00), at baseline and at the end of study. Regarding blood pressure was not significantly different among visits. We also evaluated quality of life and psychological stress associated with DM with standardized questionnaires, which were not different between the first and last visits.

## Conclusion

More frequent medical visits, provision of inputs for the treatment, including the use of PD and performing self-monitoring favored glycemic control. The glycemic goal has been achieved in this group of elderly patients with DM (with a reduction of 2.25% in average HbA1C at 6 months) with increased doses of insulin, especially regular insulin, no significant increase in hypoglycemia. Our data suggest that a change in the management of chronic decompensated elderly diabetics is required. Grants from CNPq and Fundo de Incentivo a Pesquisa do HCPA (FIPE).

